# Maintaining moderate versus lower PEEP after cardiac surgery: a propensity-scored matched analysis

**DOI:** 10.1186/s12871-024-02438-4

**Published:** 2024-02-07

**Authors:** Yi Chi, Qianling Wang, Siyi Yuan, Yutong Zhao, Huaiwu He, Yun Long

**Affiliations:** 1 State Key Laboratory of Complex Severe and Rare Disease, Department of Critical Care Medicine, Peking Union Medical College Hospital, Peking Union Medical College, Chinese Academy of Medical Sciences, 1 Shuaifuyuan, Dongcheng District, Beijing, China; 2 The First Clinical Medical College, Shanxi Medical University, 86 Xinjian South Road, Taiyuan, Shanxi China

**Keywords:** Cardiac surgery, Positive end-expiratory pressure, Mechanical ventilation, Intensive care unit, Propensity-score match

## Abstract

**Background:**

Setting positive end-expiratory pressure (PEEP) at around 5 cm H_2_O in the early postoperative period seems a common practice for most patients. It remains unclear if the routine application of higher levels of PEEP confers any meaningful clinical benefit for cardiac surgical patients. The aim of this study was to compare moderate versus conventional lower PEEP on patient-centered outcomes in the intensive care unit (ICU).

**Methods:**

This is a single-center retrospective study involving patients receiving cardiac surgery from June 2022 to May 2023. Propensity-score matching (PSM) was used to balance the baseline differences. Primary outcomes were the duration of mechanical ventilation and ICU length of stay. Secondary outcomes included PaO_2_/FiO_2_ ratio at 24 h and the need for prone positioning during ICU stay.

**Results:**

A total of 334 patients were included in the study, 102 (31%) of them received moderate PEEP (≥ 7 cm H_2_O) for the major time in the early postoperative period (12 h). After PSM, 79 pairs of patients were matched with balanced baseline data. The results showed that there was marginal difference in the distribution of mechanical ventilation duration (*p* = 0.05) and the Moderate PEEP group had a higher extubation rate at the day of T-piece trial (65 [82.3%] vs 52 [65.8%], *p* = 0.029). Applying moderate PEEP was also associated with better oxygenation. No differences were found regarding ICU length of stay and patients requiring prone positioning between groups.

**Conclusion:**

In selective cardiac surgical patients, using moderate PEEP compared with conventional lower PEEP in the early postoperative period correlated to better oxygenation, which may have potential for earlier liberation of mechanical ventilation.

**Supplementary Information:**

The online version contains supplementary material available at 10.1186/s12871-024-02438-4.

## Introduction

Cardiac surgery has a marked effect on the cardiopulmonary physiology [1]. Cardiac patients are usually at higher risk of weaning failure for mechanical ventilation and postoperative pulmonary complications including decreased lung compliance, atelectasis and infection [2, 3]. Most postoperative cardiac surgical patient are admitted to an intensive care unit (ICU) intubated and mechanically ventilated. Ventilatory support is an important component of postoperative management. Appropriate ventilator settings help to improve lung physiology, reduce postoperative pulmonary complications, and facilitate weaning from mechanical ventilation, allowing earlier ICU discharge and improved outcomes.

The effect of positive end-expiratory pressure (PEEP) setting is often underestimated in postoperative patients. Although personalized PEEP is recommended, it seems that setting PEEP at 5 cm of water (cm H_2_O) is a routine practice in most postoperative patients [4, 5], especially for those who show no signs of significant hypoxia on ICU admission. Some data suggest that higher initial PEEP after cardiac surgery (8 ~ 10 cm H_2_O vs 5 cm H_2_O) improve oxygenation, lung compliance and reduce atelectasis [4, 6, 7], yet it remains unclear if the routine application of higher PEEP results in any meaningful clinical benefit. Conflicting results exists for cardiac surgical patients on the impact of PEEP on mechanical ventilation days or ICU length of stay [4, 8–10]. In addition, postoperative hypoxemia due to atelectasis or consolidation may trigger the decision of prone positioning, which could increase clinical workload and bring some extra risks.

The aim of this retrospective study was to compare the effects of early application of moderate versus conventional lower PEEP levels in selected postoperative cardiac patients on the duration of mechanical ventilation, ICU length of stay (as primary outcomes), as well as the impact on oxygenation and the need for prone positioning during ICU stay (as secondary outcomes).

## Methods

### Settings and study population

This is a single-center retrospective study in the Department of Critical Care Medicine of a tertiary teaching hospital, with 30 surgical ICU beds. Patients admitted to the ICU after cardiac surgery during the period from June 1, 2022 to May 31, 2023 were reviewed. The exclusion criteria were patients (1) aged less than 18 years old; (2) not intubated on admission or extubated within 12 h; (3) non-survival or expected non-survival; (4) ICU length of stay over 7 days; (5) requiring fraction of inspired oxygen (FiO_2_) equal or greater than 50% on ICU admission. This study was approved by the Institutional Research and Ethics Committee of Peking Union Medical College Hospital.

### Data collection and patient grouping

Patient baseline data was retrieved from the electronic medical record system. ICU-related data were extracted from the Critical Care Monitor System in Peking Union Medical College Hospital, which recorded real-time clinical data hourly with bedside equipment. The following information was collected: demographic data, including age, sex, body mass index (BMI) and Acute Physiology and Chronic Health Evaluation (APACHE) II score; comorbidities and disease severity (hypertension, diabetes, obstructive lung disease, atrial fibrillation, chronic heart failure defined as New York Heart Association [NYHA] Classification of Heart Failure class III ~ IV, left ventricular ejection fraction [LVEF] before surgery, pulmonary hypertension); surgical information (type of surgery, cardiopulmonary bypass time, dose of epinephrine and norepinephrine); Oxygenation on ICU admission and outcomes (duration of mechanical ventilation, ICU length of stay, oxygenation after 24 h of ICU admission, prone positioning during ICU stay).

PEEP was initially set and adjusted according to the preference of physician in charge. Generally, the Lower PEEP/Higher FiO_2_ Table [11] was the reference on the initial PEEP. To take the duration of PEEP into consideration, patients were divided into two groups based on the major PEEP levels (more than 6 h) during the first 12 h of ICU admission: the Moderate PEEP group (equal or greater than 7 cm H_2_O) and the Lower PEEP group (less than 7 cm H_2_O).

### Weaning process and prone positioning criteria

In the study center, typical weaning process for patients consists of a two-step trial. A patient with eligibility for weaning (PEEP < 10 cm H_2_O, FiO_2_ < 50%, resolution of acute phase, existence of spontaneous breathing and stable hemodynamics judged by physician) would be first turned to pressure support ventilation with low-level support (PEEP 5 ~ 8 cm H_2_O, PS 6 ~ 10 cm H_2_O) for 30 min. If the patient showed no signs of respiratory distress and hemodynamic deterioration, then a T-piece trial would be followed usually for 30 min to 2 h. Patients who successfully passed the trial would be extubated and sequenced to high-flow nasal oxygen or nasal catheter. The criteria for passing the trial depended on the attending doctor and were generally stricter than existing guidelines [12, 13] to achieve a lower re-intubation rate (less than 5% in cardiac patients).

Prone positioning was advocated when the PaO_2_/FiO_2_ ratio was lower than 200 mm Hg (PEEP ≥ 8 cmH_2_O or FiO_2_ ≥ 50%) and there existed imaging evidence indicating consolidation or poor ventilation in the dorsal lungs.

### Statistical analysis

Continuous variables are expressed as median (interquartile range, IQR) and compared using Mann–Whitney U test as appropriate. Categorical variables were expressed as frequency and percentage and compared using the chi-square test or Fisher's exact test. We performed a 1:1 propensity score-matched analysis (using ‘MatchIt’ Package [14] in R software) using nearest neighbor matching without replacement and a caliper value of 0.05 to compare the Moderate PEEP group and the Lower PEEP group patients. Propensity score matching was performed based on sex, age, body mass index, APACHE II score, comorbidities, LVEF before surgery, type of surgery, cardiopulmonary bypass time, dose of epinephrine and partial pressure of arterial oxygen (PaO_2_) on ICU admission. The quality of match was assessed by examining the standardized mean differences of variables before and after matching. R software Version 4.2.2 were used for all statistical analyses, with *p* value ≤ 0.05 indicating statistical significance.

## Results

### Patient characteristics

**Fig. 1:**
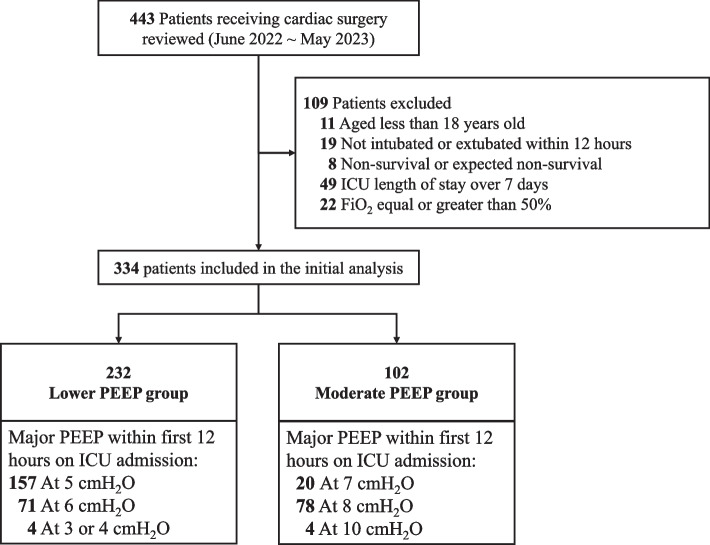
Flowchart of patient inclusion, exclusion and grouping in the study

During the study period, a total of 443 patients were reviewed and, of these, 334 were included in the initial analysis (232 [69.5%] classified as the Lower PEEP group and 102 [31.5%] as the Moderate PEEP group; Fig. 1). The patients had a median age of 57(46–65) years, and 134 (40.1%) of them were women.

**Fig. 2:**
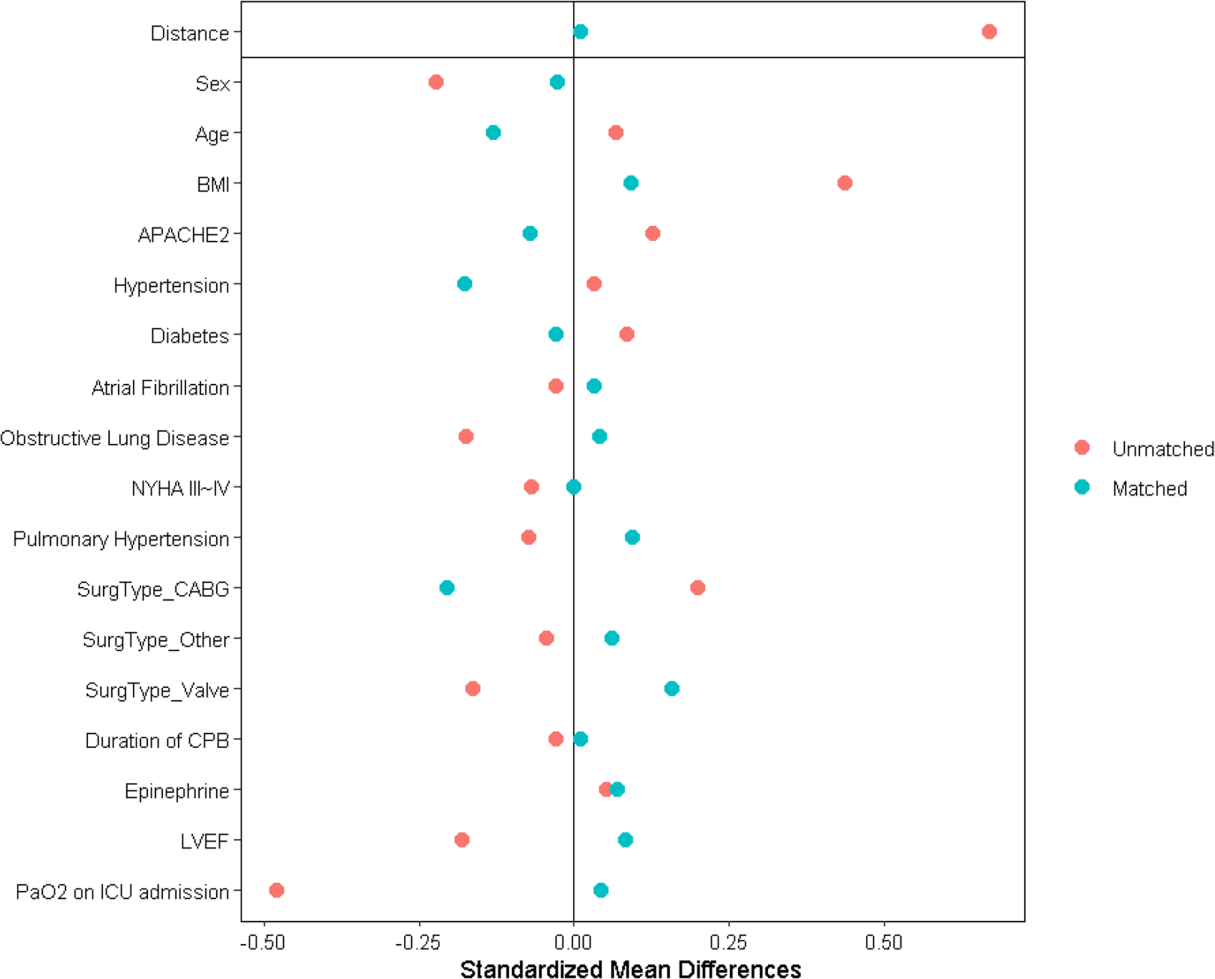
Parameters involved and visualization of propensity-scored match

The Moderate PEEP group had higher body mass index (24.8 [23.5–27.3] vs 23.7 [21.5–25.7] kg/m^2^, *p* < 0.001), lower partial pressure of oxygen (PaO_2_; 89 [75–119] vs 106 [86–129] mm Hg, *p* < 0.001) and lower PaO_2_/FiO_2_ ratio (259 [209–337] vs 311 [246–384] mm Hg, *p* < 0.001) on ICU admission. Other baseline characteristics were comparable (Table 1). After propensity score matching in a 1:1 ratio, there were 79 patients in each group, and no significant difference was found between the two groups regarding collected baseline data (Table 1; Fig. 2).

**Table 1:** Demographic and clinical data before and after propensity matching

Variable	Before Matching			After Matching		
	**Lower PEEP (*n* = 232)**	**Moderate PEEP (*n* = 102)**	***P* value**	**Lower PEEP (*n* = 79)**	**Moderate PEEP (*n* = 79)**	***P* value**
**Female, n (%)**	101 (43.5)	33 (32.4)	0.072	29 (36.7)	28 (35.4)	> 0.999
**Age, years**	57 (46–65)	58 (48–66)	0.431	59 (52–67)	56 (47–66)	0.438
**BMI, kg/m**^**2**^	23.7 (21.5–25.7)	24.8 (23.5–27.3)	** < 0.001**	24.4 (22.9–25.9)	24.6 (22.7–26.8)	0.591
**APACHE II score**	12 (9–15)	11 (9–15)	0.947	13 (10–16)	11 (9–14)	0.086
**Hypertension, n (%)**	103 (44.4)	48 (47.1)	0.741	44 (55.7)	37 (46.8)	0.340
**Diabetes, n (%)**	51 (22.0)	27 (26.5)	0.452	18 (22.8)	17 (21.5)	> 0.999
**Obstructive lung disease, n (%)**	38 (16.4)	11 (10.8)	0.245	9 (11.4)	10 (12.7)	> 0.999
**Atrial fibrillation, n (%)**	46 (19.8)	18 (17.6)	0.752	14 (17.7)	15 (19.0)	> 0.999
**Chronic heart failure, n (%)**	47 (20.3)	17 (16.7)	0.537	14 (17.7)	14 (17.7)	> 0.999
**LVEF, %**	67 (59–71)	64 (57–70)	0.083	66 (58–70)	66 (58–70)	0.697
**Pulmonary Hypertension, n (%)**	55 (23.7)	21 (20.6)	0.628	15 (19.0)	18 (22.8)	0.695
**Type of surgery, n (%)**			0.163			0.409
**Valve**	71 (30.6)	42 (41.2)		34 (43.0)	26 (32.9)	
**CABG**	58 (25.0)	23 (22.5)		19 (24.1)	21 (26.6)	
**Others**	103 (44.4)	37 (36.3)		26 (32.9)	32 (40.5)	
**Duration of CPB, min**	117 (87–148)	112 (95–138)	0.846	116 (88–144)	114 (98–137)	0.946
**On ICU admission**						
** PaO**_**2**_**, mm Hg**	106 (86–129)	89 (75–119)	** < 0.001**	89 (80–115)	90 (79–122)	0.945
** FiO**_**2**_**, %**	35 (30–40)	35 (30–40)	0.415	35 (30–40)	35 (30–40)	0.607
** P/F ratio, mm Hg**	311 (246–384)	259 (209–337)	** < 0.001**	270 (224–326)	266 (212–341)	0.921
** Crs, dyn, mL/cm H**_**2**_**O**	35.5 (30.7–42.2)	36.5 (32.1–42.9)	0.292	36.8 (30.7–42.5)	38.2 (33.3–44.2)	0.223
**12 h after ICU admission**						
** Epinephrine, mcg/kg/min**	0.00 (0.00–0.02)	0.00 (0.00–0.02)	0.863	0.00 (0.00–0.01)	0.00 (0.00–0.02)	0.504
** Norepinephrine, mcg/kg/min**	0.05 (0.00–0.18)	0.05 (0.00–0.12)	0.683	0.04 (0.00–0.18)	0.06 (0.00–0.13)	0.960

*Abbreviations BMI* Body mass index, *APACHE II* Acute Physiology and Chronic Health Evaluation, *LVEF* left ventricular ejection fraction, *CABG* coronary artery bypass graft, *CPB* cardiopulmonary bypass, *PaO*_*2*_ partial pressure of arterial oxygen, *FiO*_*2*_ fraction of inspired oxygen, *P/F, PaO*_*2*_*/FiO*_*2*_ PEEP, positive end-expiratory pressure, *Crs, dyn* dynamic compliance of respiratory system

### Outcomes

After propensity matching, there was marginal difference in the distribution of duration of mechanical ventilation (*p* = 0.050). Patients in the Moderate PEEP group had a higher rate of extubation after the first T-piece trial (65 [82.3%] vs 52 [65.8%], *p* = 0.029). No difference was found between groups regarding ICU length of stay and days from ICU admission to first T-piece trial (Table 2).

**Table 2:** Outcomes before and after propensity matching

Variable	Before Matching			After Matching		
	**Lower PEEP (*n* = 232)**	**Moderate PEEP (*n* = 102)**	***P* value**	**Lower PEEP (*n* = 79)**	**Moderate PEEP (*n* = 79)**	***P* value**
**Duration of MV, days**			0.445			**0.050**
**1**	136 (58.6)	66 (64.7)		44 (55.7)	57 (72.2)	
**2**	47 (20.3)	15 (14.7)		20 (25.3)	9 (11.4)	
≥ **3**	49 (21.1)	21 (20.6)		15 (19.0)	13 (16.5)	
**ICU admission-to-T piece, days (%)**			0.196			0.298
≤ **1**	172 (74.1)	83 (81.4)		62 (78.5)	68 (86.1)	
> 1	60 (25.9)	19 (18.6)		17 (21.5)	11 (13.9)	
**Extubation after the first T-piece trial, n (%)**	174 (74.6)	78 (76.5)	0.816	52 (65.8)	65 (82.3)	**0.029**
**ICU length of stay, days**	3 (2–4)	3 (2–4)	0.828	3 (2–5)	3 (2–4)	0.220
**PaO**_**2**_** at 24 h, mm Hg**	99 (83–120)	100 (88–127)	0.359	95 (83–106)	102 (89–132)	**0.039**
**P/F ratio at 24 h, mm Hg**	308 (262–383)	322 (251–413)	0.520	300 (253–344)	330 (254–427)	**0.048**
**Lowest PaO**_**2**_** within 24 h, mm Hg**	87 (73–102)	91 (77–105)	0.114	83 (74–98)	91 (77–112)	**0.031**
**Lowest P/F ratio within 24 h, mm Hg**	272 (221–333)	274 (234–339)	0.314	262 (217–310)	269 (234–362)	**0.046**
**Prone positioning in ICU, n (%)**	44 (19.1)	12 (11.8)	0.135	14 (17.9)	8 (10.1)	0.237

*Abbreviations*: *MV* mechanical ventilation, *ICU* intensive care unit, *PaO*_*2*_ partial pressure of arterial oxygen, *P/F, PaO*_*2*_*/FiO*_*2,*_ PEEP, positive end-expiratory pressure, *Crs, dyn* dynamic compliance of respiratory system

The Moderate PEEP group had a higher PaO_2_ (102 [89–132] vs 95 [83–106] mm Hg, *p* = 0.039) and PaO_2_/FiO_2_ ratio (300 [253–344] vs 330 [254–427] mm Hg, *p* = 0.048) at 24 h after ICU admission. The recorded lowest PaO_2_ and PaO_2_/FiO_2_ ratio within 24 h after ICU admission were also higher in the Moderate PEEP group. The proportion of patients receiving prone positioning during their ICU stay (8 [10.1%] vs 14 [17.9%] mm Hg, *p* = 0.237) did not show difference between groups (Table 2).

## Discussion

In this single-center retrospective study, we found that the early application of moderate PEEP in postoperative cardiac patients was associated with oxygenation improvement and potentially shorter duration of mechanical ventilation.

In our cohort, only around 30% of postoperative cardiac patients received moderate or higher PEEP strategy on ICU admission, others reporting this proportion around 25 ~ 34% [4, 5]. Setting lower PEEP levels (usually no more than 5 cm H_2_O) in the early postoperative period remains a common practice [15, 16], especially when the oxygenation was not or only mildly impaired, as seen in the study population. Physicians typically start considering the possibility of lung collapse and trying to increase PEEP when hypoxia was severe enough to be noticed. Atelectasis and hypoxia may contribute to weaning failure, resulting in a longer duration of mechanical ventilation. In this study, not extubating the patient after the first T-piece trial was a surrogate for weaning failure. Applying and maintaining moderate or higher PEEP levels in the early postoperative period may prevent the patients from atelectasis [17, 18].

Prone positioning may be applied to promote lung recruitment and improve oxygenation. However, turning patients prone not only increases the workload but also carries potential risks including pressure ulcers, device displacement and hemodynamic instability [19]. Additionally, surgeons may have concerns regarding the impact of prone positioning on surgical incision healing. Setting moderate or higher PEEP levels in the early postoperative period may reduce lung collapse [7], facilitate successful weaning from mechanical ventilation, and avoid the need for prone positioning as a rescue therapy. Although our data showed no statistically significant difference between groups regarding the need for prone positioning, the point estimation was in favor of the Moderate PEEP group both in the unmatched and matched cohorts.

There have been some studies with conflicting results on this topic previously, yet this study may have the following strengths: (1) The duration of PEEP was considered in patient grouping. Some studies examined the effects of intraoperative PEEP [7] or the initial PEEP at ICU [4] on patient outcomes. The time-dependent effect or the adjustment of PEEP could have been underestimated. We therefore defined that patients classified as the Moderate PEEP group should maintain at higher PEEP levels for the major time within 12 h after surgery. (2) Patient selection may be important to recognize the specific population that benefit from moderate PEEP. Patients whose primary outcomes were unlikely to be associated with their exposure PEEP levels were excluded. For example, those who did not survive or had ICU length of stay longer than 7 days were more likely to be affected by low cardiac output syndrome or the need for a second surgery rather than PEEP levels. (3) Propensity-score match with a relative strict caliper value was used to balance baseline characteristics.

There are several limitations in the present study. While the cutoff PEEP value for patient grouping may seem arbitrary and take the risk of selection bias, we originally intended to increase the PEEP differences between groups as much as possible. The effect of 1 cmH_2_O could be too little to be detected. Clinical adjustment of PEEP usually favors 2 ~ 3 cmH_2_O per step, as could be seen in the ARDSnet PEEP/FiO_2_ table and PEEP titration protocols in some randomized trials. Cutoff PEEP value of 6 or 8 cmH_2_O for grouping were also analyzed (see in Supplementary Material). The results on P/F ratio improvement favored the higher PEEP levels, while statistical significance was not found for the duration of mechanical ventilation. Lower PEEP difference between groups might mask the benefit of moderate PEEP. Second, reasons for failing to extubating patients after the first T-piece trial could be multifactorial, with the impact of PEEP may only account for a limited, yet significant part. Cardiac function could be a major potential confounding factor for weaning outcome [20]. Sometimes it is not easy to distinguish cardiac factor from lung dysfunction for weaning failure. The dose of epinephrine was recorded and balanced as an alternative parameter reflecting cardiac function. Third, measurements of static respiratory mechanics and lung volume were unavailable due to its retrospective nature of the study. Fourth, concerns on hemodynamics may be raised when using moderate or higher PEEP, but using 10 cm H_2_O PEEP was usually safe for hemodynamics [8]. In this study, dose of norepinephrine was not higher in the Moderate PEEP group. Last, propensity-score matching provides a way for narrowing basal differences between study groups, but it may not eliminate all potential confounding factors.

## Conclusion

In selective patients, using moderate PEEP compared with conventional lower PEEP in the early postoperative period after cardiac surgery correlated to shorter duration of mechanical ventilation and better oxygenation.

### Supplementary Information

**Additional file 1: Table S1.** PEEP difference between groups according to different grouping criteria.** Table S2. **Outcomes comparison between PEEP < 6 and PEEP ≥ 6 after propensity-score matching.** Table S3.** Outcomes comparison between PEEP < 8 and PEEP ≥ 8 after propensity-score matching.** Figure S1.** Correlation between set PEEP and delta P/F ratio (P/F at T24h minus P/F on admission).

## Data Availability

No datasets were generated or analysed during the current study.
