# Supplementary material for: Maintaining moderate versus lower PEEP after cardiac surgery: a propensity-scored matched analysis
Source: BMC Anesthesiol. 2024 Feb 7;24:55. doi: 10.1186/s12871-024-02438-4 (PMC10848339; doi:10.1186/s12871-024-02438-4)

**Supplementary material**

**Table S1.** PEEP difference between groups according to different grouping criteria

| PEEP cutoff value for Grouping | The Lower PEEP group | | The Moderate PEEP group | | Mean PEEP difference between groups after PSM |
| --- | --- | --- | --- | --- | --- |
|  | Possible PEEP value | Mean PEEP value after PSM | Possible PEEP value | Mean PEEP value after PSM |  |
| **7** | 3,4,5,6 | **5.2** | 7,8,10 | **8.0** | **2.8** |
| **6** | 3,4,5 | **5.0** | 6,7,8,10 | **7.0** | **2.0** |
| **8** | 3,4,5,6,7 | **5.4** | 8,10 | **8.1** | **2.7** |

**Table S2.** Outcomes comparison between **PEEP < 6 and PEEP ≥ 6** after propensity-score matching

| Variable | After Matching | | |
| --- | --- | --- | --- |
|  | **PEEP < 6**  **(n = 115)** | **PEEP ≥ 6**  **(n = 115)** | **P value** |
| **Duration of MV, days** |  |  | 0.407 |
| **1** | 65 (56.5%) | 71 (61.7%) |  |
| **2** | 26 (22.6%) | 18 (15.7%) |  |
| **≥ 3** | 24 (20.9%) | 26 (22.6%) |  |
| **ICU length of stay, days** | 3 (2-4) | 3 (2-4) | 0.705 |
| **PaO_2_ at 24 hours, mm Hg** | 101 (82-120) | 100 (90-124) | 0.413 |
| **P/F ratio at 24 hours, mm Hg** | 320 (259-384) | 320 (273-397) | 0.424 |
| **Lowest PaO_2_ within 24 hours, mm Hg** | 87 (73-99) | 89 (76-103) | 0.276 |
| **Lowest P/F ratio within 24 hours, mm Hg** | 265 (220-325) | 275 (234-336) | 0.167 |
| **Prone positioning in ICU, n (%)** | 22 (19.5%) | 19 (16.5%) | 0.684 |

**Table S3.** Outcomes comparison between **PEEP < 8 and PEEP ≥ 8** after propensity-score matching

| Variable | After Matching | | |
| --- | --- | --- | --- |
|  | **PEEP < 8**  **(n = 72)** | **PEEP ≥ 8**  **(n = 72)** | **P value** |
| **Duration of MV, days** |  |  | 0.549 |
| **1** | 44 (61.1%) | 44 (61.1%) |  |
| **2** | 9 (12.5%) | 13 (18.1%) |  |
| **≥ 3** | 19 (26.4%) | 15 (20.8%) |  |
| **ICU length of stay, days** | 3 (2-5) | 3 (2-4) | 0.408 |
| **PaO_2_ at 24 hours, mm Hg** | 95 (82-113) | 100 (91-124) | 0.065 |
| **P/F ratio at 24 hours, mm Hg** | 283 (246-346) | 322 (259-403) | **0.045** |
| **Lowest PaO_2_ within 24 hours, mm Hg** | 83 (70-98) | 91 (78-103) | **0.031** |
| **Lowest P/F ratio within 24 hours, mm Hg** | 246 (216-301) | 282 (239-335) | **0.019** |
| **Prone positioning in ICU, n (%)** | 17 (23.6%) | 7 (9.7%) | **0.044** |

**Figure S1.** Correlation between set PEEP and delta P/F ratio (P/F at T24h minus P/F on admission).


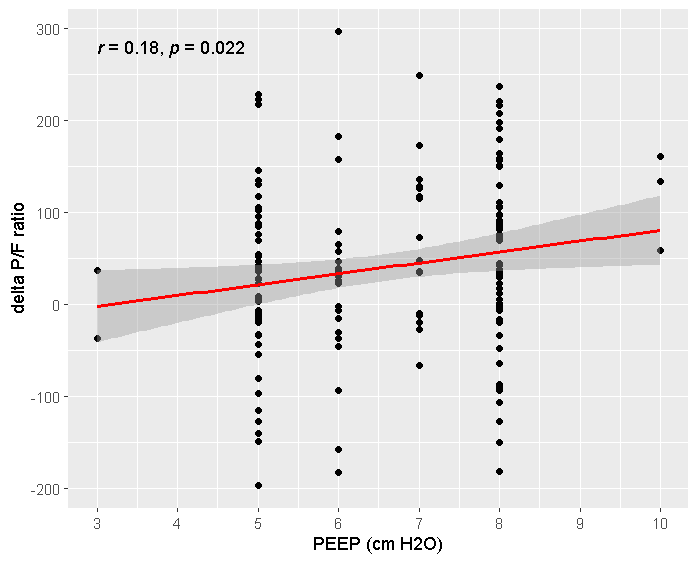

Supplement: Supplementary file 1 — Additional file 1: Table S1. PEEP difference between groups according to different grouping criteria. Table S2. Outcomes comparison between PEEP < 6 and PEEP ≥ 6 after propensity-score matching. Table S3. Outcomes comparison between PEEP < 8 and PEEP ≥ 8 after propensity-score matching. Figure S1. Correlation between set PEEP and delta P/F ratio (P/F at T24h minus P/F on admission). [file 12871_2024_2438_MOESM1_ESM.docx]
